# A network-based approach to discover diagnostic metabolite markers associated with depressive features for major depressive disorder

**DOI:** 10.3389/fpsyt.2025.1610520

**Published:** 2025-06-06

**Authors:** Yuzhen Zheng, Duan Zeng, Ying Tian, Siyuan Li, Shen He, Huafang Li

**Affiliations:** ^1^ Shanghai Mental Health Center, Shanghai Jiao Tong University School of Medicine, Shanghai, China; ^2^ Clinical Research Center, Affiliated Hospital of Shandong University of Traditional Chinese Medicine, Jinan, China; ^3^ Department of Psychiatry, Shanghai Mental Health Center, Shanghai Jiao Tong University School of Medicine, Shanghai, China; ^4^ Shanghai Key Laboratory of Psychotic Disorders, Shanghai, China

**Keywords:** major depressive disorder, metabolomics, biomarkers, machine-learning, WGCNA

## Abstract

**Background:**

Despite the high prevalence of major depressive disorder (MDD), current diagnostic methods rely on subjective clinical assessments, highlighting the need for biomarkers. This study aimed to investigate plasma metabolite signatures in patients with MDD compared with healthy controls (HC) and to identify diagnostic biomarkers associated with depressive features.

**Methods:**

A total of 99 patients with MDD and 50 HC were included in this study from a study cohort. Targeted plasma metabolomics was employed to quantify metabolites across diverse biochemical classes. Weighted gene co-expression network analysis (WGCNA) was performed to construct metabolite networks and identify modules and metabolites associated with depressive features. Diagnostic models were developed based on the identified hub metabolites, using six supervised machine-learning algorithms. Model interpretability was enhanced through the application of the SHapley Additive exPlanations (SHAP) algorithm.

**Results:**

Pathways such as biosynthesis of phenylalanine, tyrosine and tryptophan, glutathione metabolism, and arginine and proline metabolism were significantly enriched in the comparison of metabolic profiles between the MDD and HC groups. Seven hub metabolites were identified as the biomarker signatures that effectively discriminate the MDD and HC groups. Among these metabolites, one sphingomyelin (SM (OH) C16:1), one hexosylceramide (HexCer(d18:1/24:1)), one phosphatidylcholine (PC aa C40:6), and one cholesteryl ester (CE(20:4)) were positively associated with the depression severity, sadness/depressive mood, and other depressive features, while methionine, arginine, and tyrosine showed negative correlation. The deep neural network model incorporating these seven biomarkers achieved the highest diagnostic performance, with an area under the curve (AUC) of 0.803 (95% CI, 0.643–0.962).

**Conclusion:**

We identified a novel signature of seven biomarkers for constructing an explainable diagnostic model that effectively discriminates between the MDD and HC groups. These biomarkers were associated with depressive symptoms. The findings provide new insights into the biological diagnosis of MDD.

**Clinical Trial Registration:**

https://clinicaltrials.gov/search?cond=NCT04518592.

## 1 Introduction

Major depressive disorder (MDD) is a complex psychiatric disease, affecting approximately 6% of the global population annually (1). Despite extensive research, the exact mechanisms underlying MDD remain incompletely understood. Currently, no clinically applicable diagnostic biomarkers have been established for MDD, and its diagnosis largely depends on clinicians’ subjective assessments. Due to the lack of objective diagnostic methods, fewer than half of patients with MDD receive effective treatment (2). Consequently, research on biomarkers for MDD holds substantial clinical significance.

Metabolic dysregulation has been recognized as one of the underlying etiological factors in MDD (3, 4). Previous studies have explored a series of blood metabolites with potential diagnostic value for MDD. Metabolites involved in the neurotransmitter system, such as γ-aminobutyric acid (GABA), dopamine, tyramine, and kynurenine, have the potential to become biomarkers for MDD (5). Lipid metabolites derived from phosphatidylcholine, phosphatidylethanolamine, sphingomyelin, and triacylglycerol have been reported as potential biomarkers (6). Altered plasma levels of amino acids, including proline, serine, arginine, phenylalanine, and glycine have been observed in patients with MDD (7).

Previous studies have primarily focused on specific metabolite classes, potentially overlooking interclass metabolic interactions (5, 8). Comprehensive metabolomic studies examining a broad spectrum of metabolite classes in MDD are still limited. Moreover, although many metabolic biomarkers have been proposed, their relationships with clinical features remain inadequately characterized. Given the high clinical heterogeneity in patients with MDD (9), the identification of biomarkers associated with clinical features is essential for advancing precision diagnosis.

Metabolomic analyses generate vast amounts of data, and investigating univariate associations between MDD and metabolites may not be sufficient for diagnosing the disease. Weighted gene co-expression network analysis (WGCNA), as a powerful network-based approach, was developed to effectively identify modules associated with sample traits (10). The application of WGCNA for MDD research has expanded from transcriptomics to other high-throughput omics data, including metabolomics (11). For instance, a clinical study employed WGCNA to identify metabolic signatures associated with antidepressant response in lipoprotein profiles (12). Another study using WGCNA found that the acylcarnitine module was inversely associated with depressive symptomatology (13).

The WGCNA is a valuable tool for identifying biomarkers associated with disease symptoms. Meanwhile, machine learning can efficiently process high-dimensional and complex datasets, enabling the extraction of hub features from the modules identified by WGCNA (14, 15). The biological context provided by WGCNA, combined with the computational advantages of machine learning, could offer robust insights for investigating complex diseases such as MDD (16). However, studies applying WGCNA combined with multiple machine learning approaches to analyze metabolomics data for MDD diagnostic model development are still limited.

Therefore, this study aimed to use WGCNA to identify metabolomic signatures associated with depressive features, such as overall depression severity and depressive mood, and to explore diagnostic biomarkers for MDD using various machine learning techniques. Correlations between metabolite modules and depressive features were calculated within the metabolite networks. Diagnostic models were developed based on hub metabolites. Six machine-learning algorithms were applied to enhance the diagnostic performance of combined metabolites. Model interpretability was enhanced through the application of the SHapley Additive exPlanations (SHAP) algorithm.

## 2 Materials and methods

### 2.1 Study design and data

In our previous Integrated Module of Multidimensional Omics for Peripheral Biomarkers (iMORE) cohort study, we profiled plasma metabolites associated with MDD by the targeted metabolomics approach (17). To further investigate metabolic diagnostic biomarkers for MDD, we included 99 patients with MDD and 50 healthy controls (HC) from the iMORE cohort. This subgroup had complete clinical and metabolomic data at baseline, constituting the largest available subset within the cohort. The iMORE study is a prospective, observational cohort study conducted at the Shanghai Mental Health Center (17). The study protocol was approved by the Institutional Review Board of Shanghai Mental Health Center (approval number 2020-87). Clinical and metabolomic data were collected between December 2020 and September 2021 at the Shanghai Mental Health Center.

### 2.2 Inclusion and exclusion criteria

The inclusion criteria for patients with MDD were as follows: (1) aged 18–65 years; (2) diagnosed with MDD (first or recurrent episode) according to the DSM-5 criteria; (3) Hamilton Depression Rating Scale (HAMD-17) total score of 20 or higher at screening; (4) Montgomery-Asberg Depression Rating Scale (MADRS) total score of 24 or higher at screening; and (5) provided informed consent. The exclusion criteria primarily included a diagnosis of any current Axis I mental disorder other than MDD or identification of serious suicide risk or suicidal thoughts (score >3 on item 10 of the MADRS). The comprehensive exclusion criteria are detailed in the protocol (17). Healthy adults with matched age ranges and sex ratios were recruited as controls, and individuals with a family history or personal history of mental disorders were excluded.

### 2.3 Depressive symptom assessments

Depressive features were primarily quantified using individual item scores from the MADRS and HAMD-17 scales. Suicide-related features were not analyzed, as patients with high suicide risk had been excluded during the recruitment phase. Pearson correlation analysis was performed to evaluate the associations between metabolites and depressive features.

### 2.4 Metabolite detection in plasma

Plasma samples from patients with MDD and HC were preserved for targeted metabolomics assays via the MxP^®^ Quant 500 kit (BIOCRATES Life Science AG, Austria) on an ultra-performance liquid chromatography (UPLC)/MS/MS system [ExionLC UPLC (Sciex), QTRAP 6500+ triple quadrupole/linear ion trap MS/MS (Sciex)]. This system enables the quantitative and semiquantitative determination of up to 630 endogenous and microbially derived metabolites (**Supplementary Table S1**). The plasma samples were analyzed following the manufacturer’s protocol, with each plate containing four replicates of the QC pool, which consisted of 10 pooled human plasma samples. The samples were analyzed under blinded diagnostic conditions.

### 2.5 Multivariate analysis of metabolites

Multivariate analysis was performed to identify differential metabolites. Orthogonal partial least squares discriminant analysis (OPLS-DA) was applied to discriminate variation in the metabolomic profiles between the MDD and HC groups. Variable importance in projection (VIP) scores of each metabolite were calculated, which represents the contribution and explanatory power in distinguishing the two groups. The robustness of the OPLS-DA model was evaluated using R²Y and Q² values and further validated through a 200-time permutation test. Differences in metabolite levels between the two groups were assessed using Student’s t-tests and fold change analysis. Metabolites were considered significantly different based on commonly used thresholds of VIP > 1 and *p* < 0.05 (18).

### 2.6 Metabolite co-expression network analysis

WGCNA was conducted on all metabolites using the *WGCNA* package in R to identify modules associated with depressive features. Based on the soft threshold power and mean connectivity, the weighted coefficient β was set to seven to approximate a scale-free metabolite network (**Supplementary Figure S1**). A hierarchical clustering dendrogram was constructed based on the adjacency matrix and topological overlap matrix to identify metabolite modules. Significant modules were identified by calculating Pearson’s correlations between module eigengenes (MEs) and depressive features. Metabolites in the significant modules with a module membership (MM) > 0.6 and gene significance (GS) > 0.2 were identified as candidate hub metabolites. The intersection of differentially expressed metabolites and candidate hub metabolites was defined as the final hub metabolite set. These metabolites were subsequently evaluated as potential diagnostic biomarkers for MDD.

### 2.7 Metabolic pathway analysis

Metabolic pathway analysis was performed using the *MetaboAnalystR* package in R. Quantitative Metabolite Set Enrichment Analysis (MSEA) was conducted on all metabolites to identify pathways associated with MDD. Additionally, pathway enrichment analysis was performed on the differentially expressed metabolites using the hypergeometric test. Pathways with *p* less than 0.05 were considered statistically significant.

### 2.8 Diagnostic model construction and evaluation

Diagnostic models were developed using the identified hub metabolites as predictive features. The dataset of 149 samples was randomly split into a training set (70%) and a testing set (30%) for model development and validation, respectively. Feature selection was performed using the elastic net algorithm to reduce multicollinearity among features in the training set. Six supervised machine learning algorithms—ridge regression, naive Bayes, support vector machine (SVM), random forest (RF), extreme gradient boosting (XGBoost), and deep neural network (DNN)—were employed to construct diagnostic models. All models were trained using the same training dataset. Both feature selection and model training were performed using five-fold cross-validation for hyperparameter tuning within the training set. Model performance was evaluated by AUC, accuracy, F1 score, and recall. Models’ predictive calibration was evaluated using the Hosmer–Lemeshow test and calibration curves.

### 2.9 Model interpretation

The SHAP analysis was used to interpret the models by evaluating the contribution of each feature for each sample to classification performance (19). Higher mean absolute SHAP values indicated greater feature importance in the model. SHAP is particularly useful for interpreting complex models.

### 2.10 Statistical analysis

All statistical analyses were performed using the R software (4.4.1). Metabolites with more than 30% missing values were excluded, and only those quantified in at least 50% of the samples were retained for analysis. Missing metabolite values were imputed using the k-nearest neighbor method. Metabolite concentration data were normalized and log-transformed. Demographic characteristics and clinical scale scores were compared between the MDD and HC groups using appropriate statistical tests. Continuous variables were analyzed using t-tests for normally distributed data and the Mann–Whitney U test for non-normally distributed data. For metabolites, the odds ratio (OR) and 95% confidence interval (CI) for MDD risk were calculated using univariate logistic regression. An OR of less than 1 indicates a protective factor, while an OR of greater than 1 indicates a risk factor. A two-tailed *p <*0.05 was considered statistically significant.

## 3 Results

### 3.1 Participant characteristics

**Figure 1:**
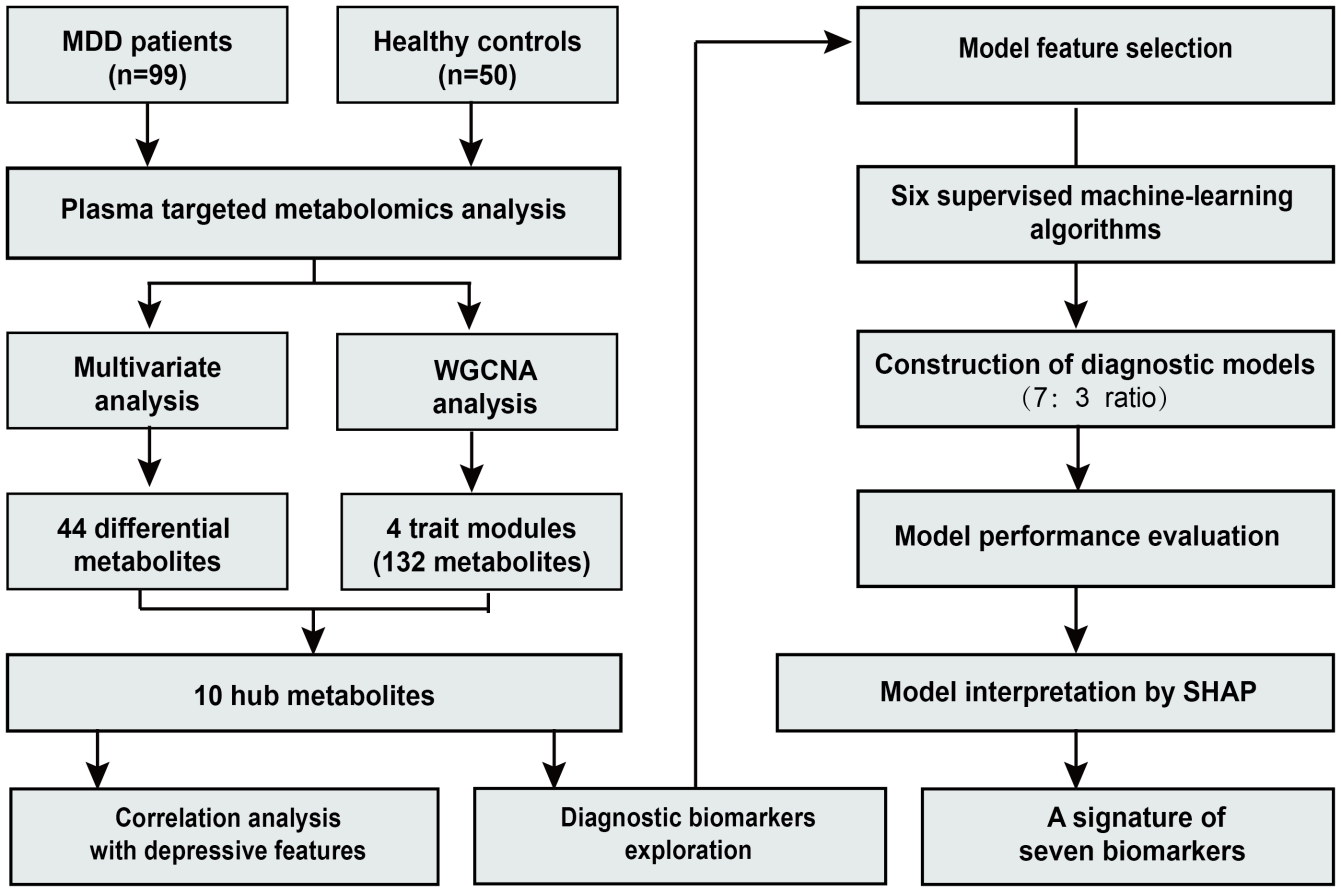
Flowchart of the study.

An overview of the study design and workflow is presented in **Figure 1**. A total of 99 patients with MDD and 50 HC were included in the analysis. The mean age (SD) of all participants was 27.1 (8.2) years. Among them, 29.5% were male and 70.5% were female. In the MDD group, depressive symptom severity was assessed using depression scales, with a mean (SD) score of 32.6 (5.6) on the MADRS, 25.6 (4.2) on the HAMD-17, and 24.1 (6.6) on the Hamilton Anxiety Rating Scale (HAMA-14). No significant differences were observed in age, sex, or BMI between the two groups. Detailed demographic and clinical characteristics of the participants have been described previously (17).

### 3.2 Identification of differential metabolites

**Figure 2:**
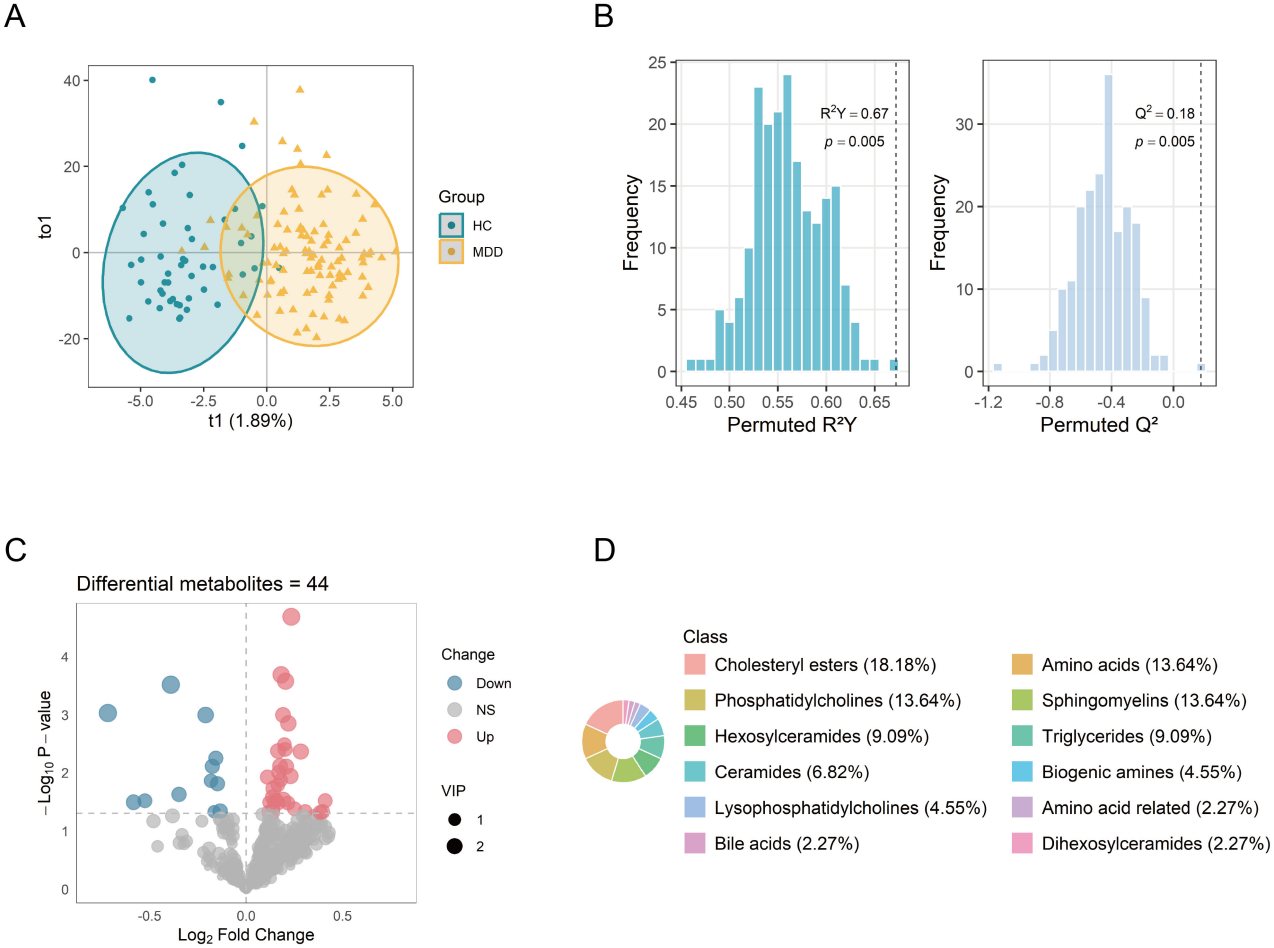
Identification of differential metabolites between MDD patients and healthy controls. **(A)** OPLS-DA score plot showing distinct metabolic profiles between the two groups. **(B)** Permutation test (n = 200) evaluating the robustness of the OPLS-DA model. **(C)** Volcano plot of 427 metabolites, with point size indicating the corresponding VIP values. **(D)** Biochemical classes of the 44 differential metabolites. OPLS-DA, orthogonal projections to latent structure-discriminant analysis; VIP, variable importance in projection.

Following data preprocessing, a total of 427 metabolites were successfully quantified for further analysis. The OPLS-DA analysis revealed differences in the metabolic profiles between the MDD and HC groups (**Figure 2A**). VIP values of metabolites were calculated by the OPLS-DA model. Metabolites with higher VIP values showed greater importance in discriminating between the two groups. A total of 158 metabolites exhibited VIP scores >1. The robustness of the model was demonstrated by the permutation testing (n=200) with R²Y = 0.67, Q² = 0.18, and *p* < 0.01 (**Figure 2B**). Metabolite levels between the groups were compared by the Student’s t-test. A total of 44 differential metabolites were identified (VIP >1, *p* < 0.05), including 32 upregulated and 12 downregulated metabolites. The differential metabolites were visualized via the volcano plot (**Figure 2C**). The major classes of these differential metabolites included cholesteryl esters (CEs, 18.18%), amino acids (13.64%), phosphatidylcholines (PCs, 13.64%), and sphingomyelins (SMs, 13.64%) (**Figure 2D**).

### 3.3 Pathway analysis of metabolites

**Figure 3:**
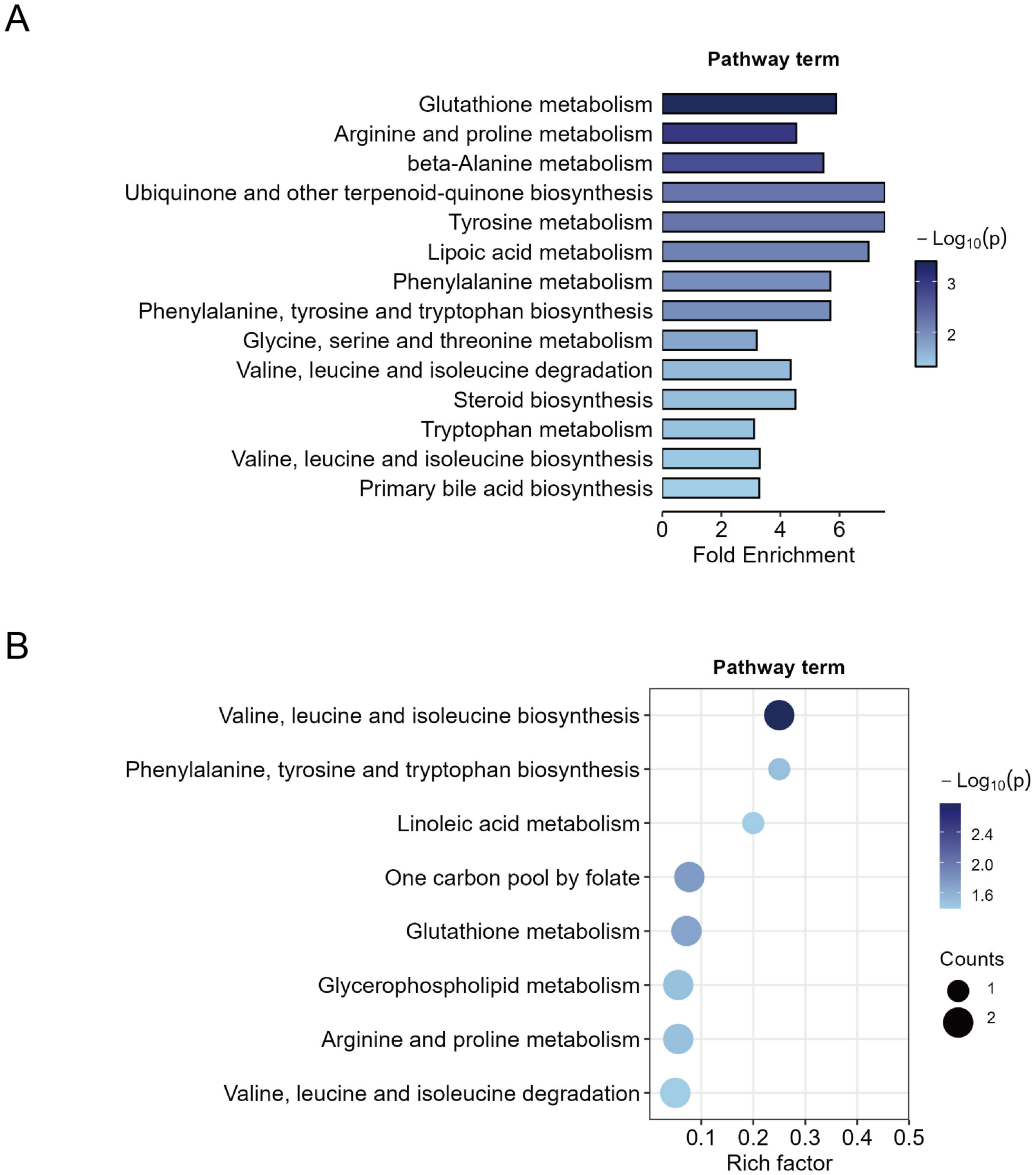
Metabolic pathway enrichment analysis. **(A)** Quantitative metabolite set enrichment analysis of 427 metabolites between MDD patients and healthy controls. **(B)** Pathway enrichment of 44 identified differential metabolites.

First, MSEA was performed on all 427 metabolites based on their fold changes to identify enriched pathways between the MDD and HC groups. The enrichment test was based on metabolite sets in KEGG (Kyoto Encyclopedia of Genes and Genomes) human metabolic pathways. A total of 14 significant metabolic pathways were identified (*p* < 0.05) (**Figure 3A**). Pathways with high fold enrichment included glutathione metabolism, arginine and proline metabolism, and tyrosine metabolism. To obtain more specific pathways with function, we performed KEGG pathway enrichment analysis on the 44 differential metabolites and identified eight significant metabolic pathways (*p* < 0.05). The top three pathways included valine, leucine and isoleucine biosynthesis, linoleic acid metabolism, and phenylalanine, tyrosine and tryptophan biosynthesis (**Figure 3B**). Four important metabolic pathways were identified both in the MSEA and KEGG pathways enrichments: biosynthesis or degradation of valine, leucine and isoleucine, biosynthesis of phenylalanine, tyrosine and tryptophan, glutathione metabolism, and arginine and proline metabolism. The findings suggest that dysregulation of these metabolic pathways is closely associated with MDD.

### 3.4 WGCNA identifies depressive features-associated modules

**Figure 4:**
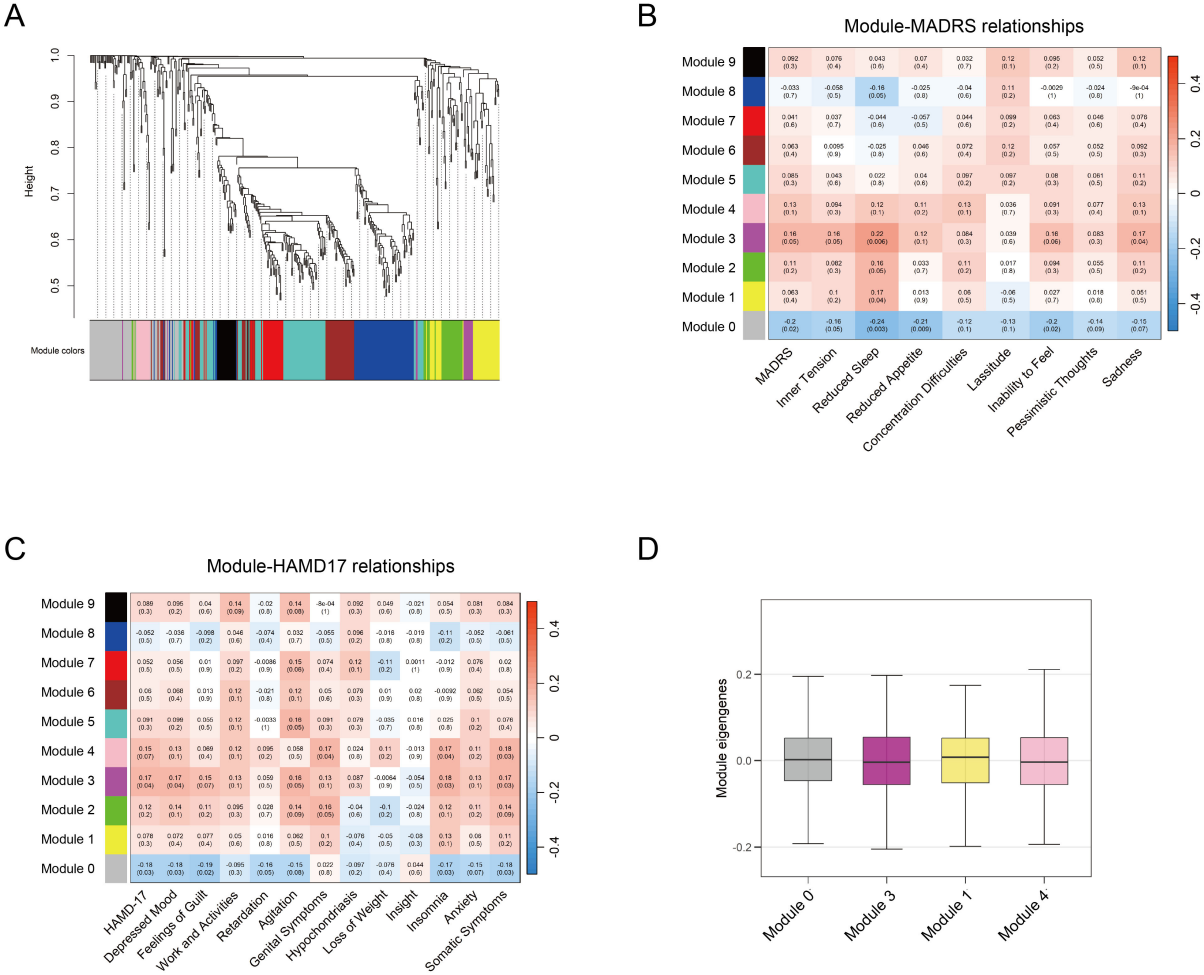
WGCNA analysis of metabolites. **(A)** Module clustering of 427 metabolites using WGCNA, with different colors representing distinct modules. **(B-C)** Module-trait correlations between module eigengenes and depression rating scales (MADRS and HAMD-17, respectively). Significant correlations are defined as *p* < 0.05 **(D)** Average MEs of the identified four modules associated with depression traits. WGCNA, weighted gene co-expression network analysis; MADRS, Montgomery-Asberg Depression Rating Scale; HAMD-17, Hamilton Depression Rating Scale; MEs, module eigengenes.

WGCNA was performed to identify co-expression modules associated with depressive features among 427 metabolites. Depressive features were quantified using individual item scores from the MADRS and HAMD-17 scales. To avoid redundancy, the sadness features (items 1, 2) in MADRS were combined. Features of insomnia (items 4, 5), anxiety (items 10, 11), and somatic symptoms (items 12, 13) in HAMD-17 were combined respectively. The analysis identified ten distinct metabolite co-expression modules, with each represented by a unique color (**Figure 4A**). The MEs, defined as the first principal component of each module, were calculated. The correlation between module MEs and depressive features was evaluated, and four significant modules (Modules 0, 1, 3, and 4) were identified (*p* < 0.05) (**Figures 4B–D**). These four modules were significantly associated with multiple depressive features, including depression severity, sadness, insomnia, and somatic symptoms.

The metabolite interaction networks within the significant modules were further analyzed using Cytoscape (version 3.7.2) with the Molecular Complex Detection (MCODE) plugin. The primary networks were visualized in **Supplementary Figure S2**. Pathway enrichment in these modules identified 13 significant metabolic pathways (**Supplementary Figure S3**). Furthermore, 23 candidate hub metabolites were identified from these four modules with the thresholds of MM > 0.6 and GS > 0.2, representing their association with the respective modules and depressive features.

### 3.5 Signatures of the ten hub metabolites

**Figure 5:**
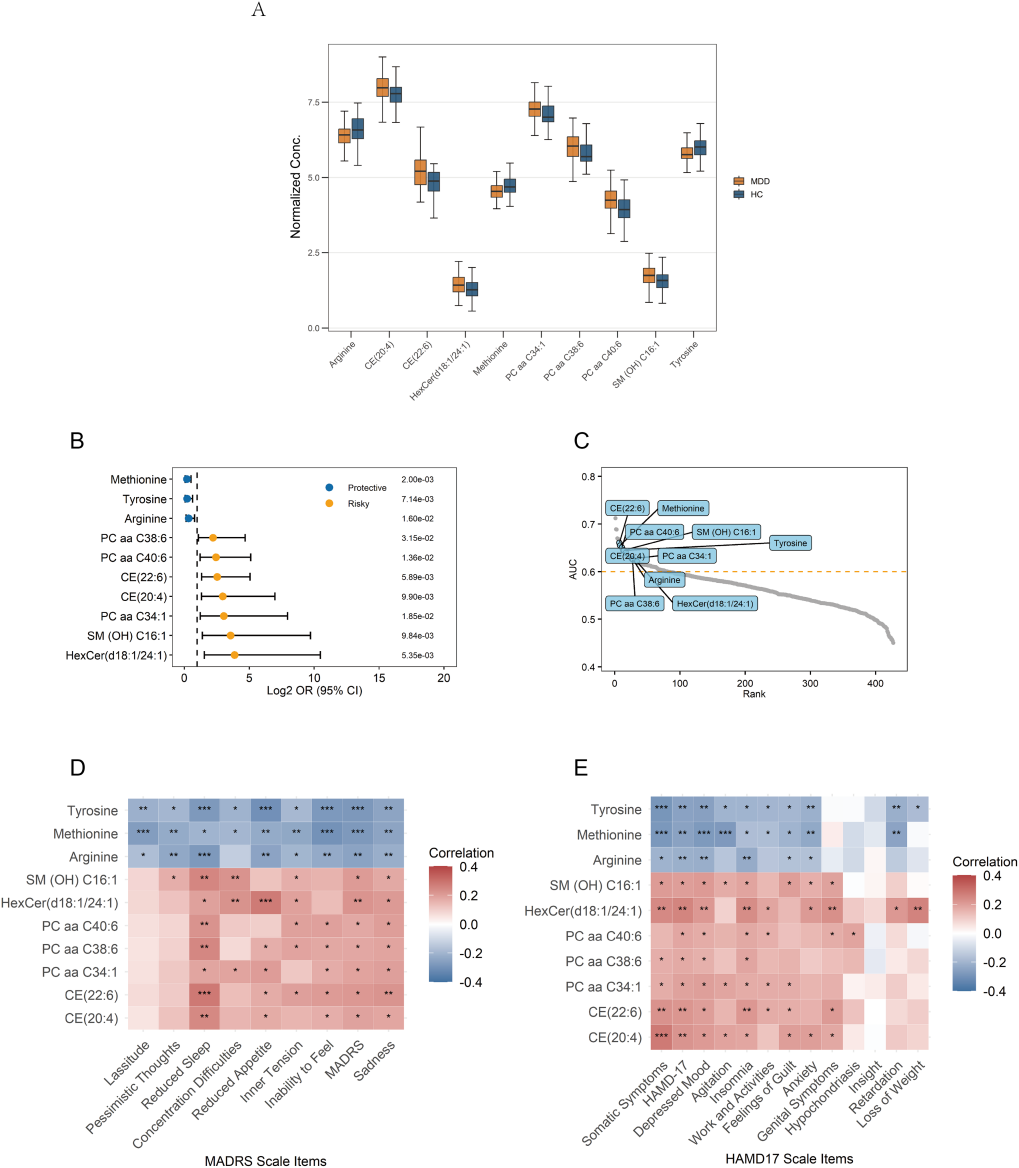
Signature of the 10 identified hub metabolites. **(A)** Normalized concentrations of the hub metabolites in MDD patients and healthy controls. **(B)** Univariate logistic regression analysis of the hub metabolites. **(C)** Ranking of AUC values for the hub metabolites among all metabolites in ROC analysis. **(D, E)** Correlations between the hub metabolites and depression rating scales; Correlations are calculated using Pearson’s correlation, with significance levels indicated as ***p < 0.001, **p < 0.01, and *p < 0.05. AUC, Area Under the Curve; ROC, Receiver Operating Characteristic.

A total of ten hub metabolites were identified by intersecting the 44 differentially expressed metabolites with the 23 candidate hub metabolites. These included a hexosylceramide (HexCer(d18:1/24:1)), a sphingomyelin (SM (OH) C16:1), three phosphatidylcholines (PC aa C34:1, PC aa C40:6, PC aa C38:6), two cholesteryl esters (CE(20:4), CE(22:6)), and three amino acids (arginine, tyrosine, methionine). The normalized concentration of these metabolites in the MDD and HC groups is shown in **Figure 5A**. Univariate logistic regression analysis was conducted to estimate the risk for MDD in individual metabolites. The results suggested that methionine, tyrosine, and arginine were protective factors, whereas the other seven hub metabolites were risk factors (**Figure 5B**). Their diagnostic potential was further assessed using ROC analysis. The AUC values of these metabolites ranged from 0.621 to 0.660, with a high rank among all the metabolites, indicating their potential diagnostic value (**Figure 5C**).

### 3.6 Correlation with depressive features

Pearson correlation analysis was conducted to examine associations between the identified hub metabolites and depressive symptom features. Arginine, tyrosine, and methionine exhibited significant negative correlations with multiple depressive features, such as depression severity and sadness/depressive mood. In contrast, the remaining seven metabolites showed significant positive correlations, indicating their association with increased symptom severity (**Figures 5D, E**). Among these metabolites, the following metabolites exhibited the more significant positive correlations (*p*<0.01): HexCer(d18:1/24:1), CE(20:4), and CE(22:6) positively correlated with total depression scores and somatic symptoms; HexCer(d18:1/24:1) and CE(22:6) positively correlated with sadness/depressed mood; SM(OH)C16:1, PC aa C40:6, PC aa C38:6, CE(20:4), CE(22:6), and HexCer(d18:1/24:1) positively correlated with insomnia/reduced sleep.

### 3.7 Construction and evaluation of diagnostic models

To evaluate their diagnostic potential, machine-learning models were constructed using the ten identified hub metabolites. The collinearity among these metabolites was evaluated in **Supplementary Figure S4**. To identify important variables and minimize their collinearity, the elastic net algorithm was used for feature selection in the training set (**Supplementary Figure S5**). Ultimately, seven metabolites (SM (OH) C16:1, HexCer(d18:1/24:1), methionine, PC aa C40:6, arginine, CE(20:4), and tyrosine) were selected as features for model construction. Six machine-learning algorithms were applied to the training set to construct diagnostic models. The detailed parameters and primary R packages used for all model training are provided in **Supplementary Table S2**.

**Figure 6:**
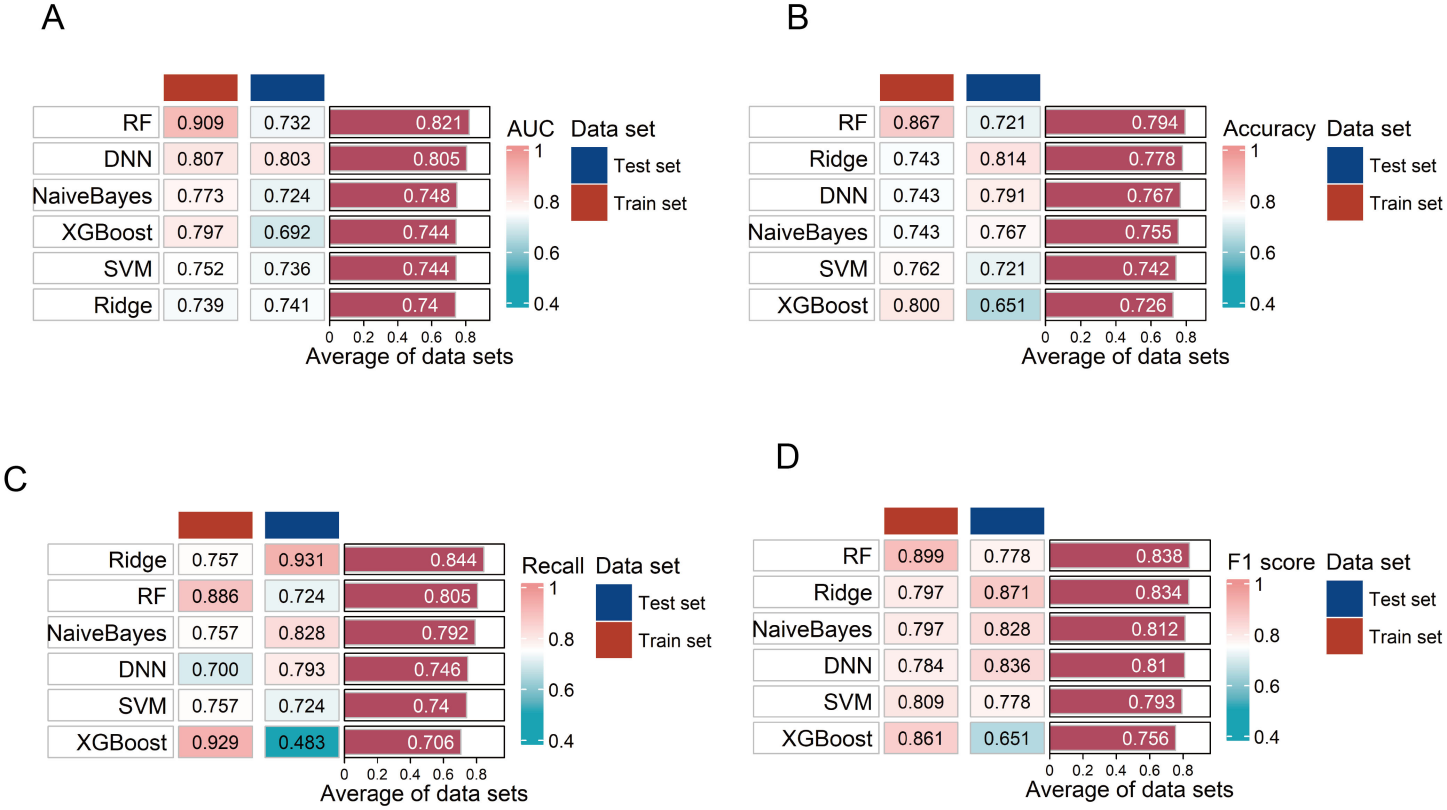
Evaluation of the six diagnostic models. **(A–D)** Evaluation of six diagnostic model performances on training and testing sets using area under the curve (AUC), accuracy, F1 score, and recall. RF, random forest; SVM, support vector machine; DNN, deep neural network.

Compared to other models, the DNN model was identified as the optimal diagnostic model with the highest AUC in the testing set (**Figure 6A**). In the ROC curve, the AUC of the model was 0.803 (95% CI: 0.643–0.962), sensitivity was 79.3%, and specificity was 78.6% (**Supplementary Figure S6**). The accuracy of the model was 0.791, the recall was 0.793, and the F1 score was 0.836 (**Figures 6B–D**). The model also demonstrated a good calibration with a *p*-value of 0.090 on the Hosmer-Lemeshow test. The DNN model consisted of two hidden layers, each with 15 neurons, using the rectifier activation function with a dropout rate of 10.0% in the hidden layers (20). The performance of the other five models in distinguishing the patients from HC is shown in **Figures 6A–D**.

### 3.8 Model interpretability

**Figure 7:**
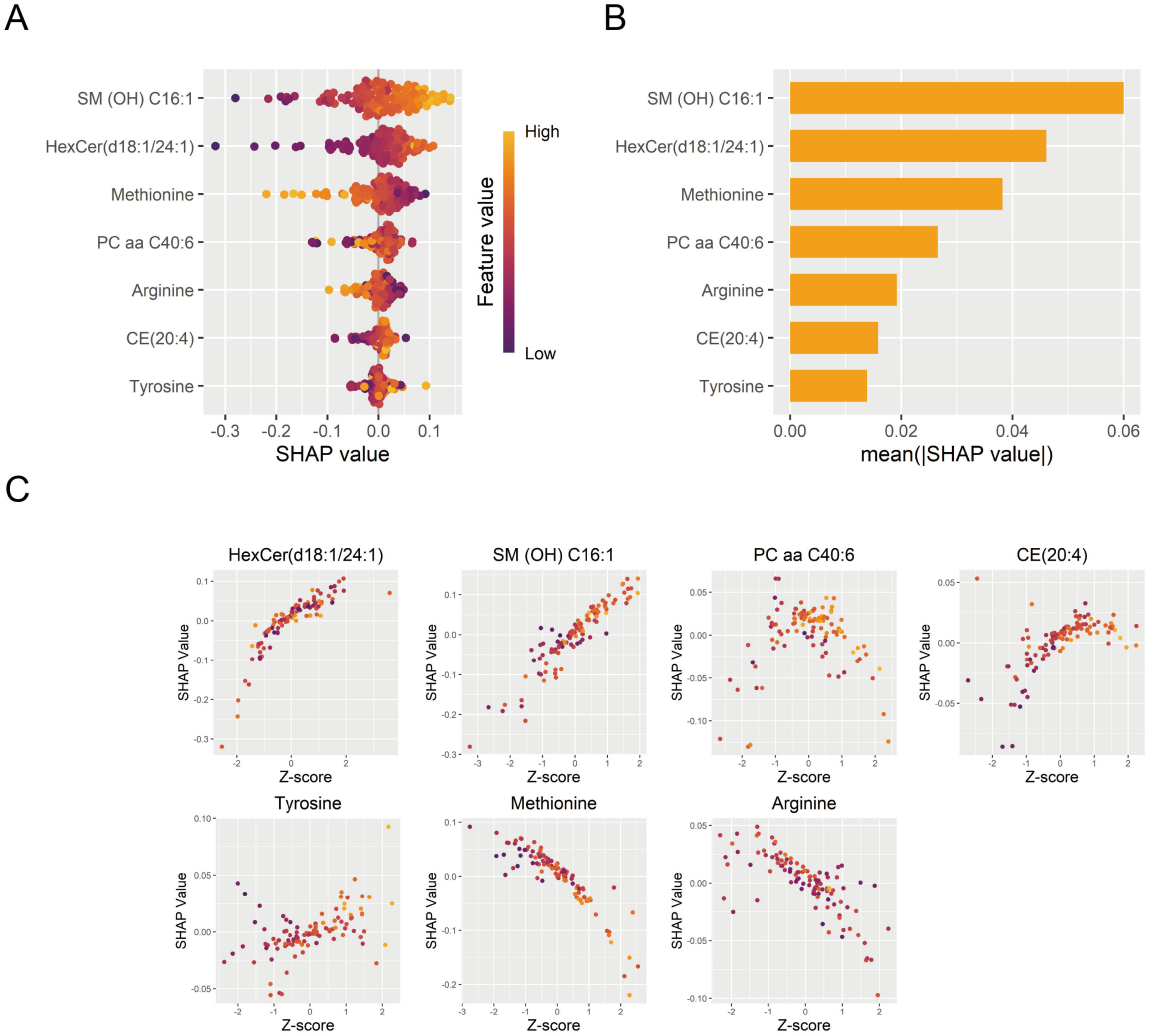
Interpretation of the DNN diagnostic model. **(A)** Contribution of each diagnostic marker from individual samples to the DNN diagnostic model’s performance. **(B)** Importance ranking of model features based on mean absolute SHAP values. **(C)** The effects of diagnostic marker expression on SHAP values. DNN, deep neural network; SHAP, SHapley Additive exPlanations.

The DNN model was interpreted using the SHAP algorithm to evaluate the contribution of each feature to its diagnostic predictions in the training set. SHAP quantified the directional contribution of features to the prediction, where positive values indicated increased diagnostic possibility for MDD and negative values indicated a higher possibility for HC. A higher mean absolute SHAP value indicated greater importance of features to the model. SM (OH) C16:1 was identified as the most important contributor to the model (**Figures 7A, B**). Furthermore, the relationship between standardized metabolite levels (Z-scores) and the SHAP values was displayed in **Figure 7C**, illustrating how a feature influenced diagnostic predictions.

## 4 Discussion

The rising global prevalence of MDD highlights the urgent need for the development of diagnostic biomarkers (21). Accumulated evidence indicates that metabolic dysregulation has been implicated in the pathological mechanism of MDD. Metabolomics serves as a powerful tool to investigate systemic metabolic alterations and identify potential biomarkers for MDD.

Through targeted metabolomics, we identified the metabolic profiles from various classes between patients with MDD and HC. Cholesteryl esters, amino acids, phosphatidylcholines, and sphingomyelin were the primary differential metabolites. Pathway enrichment highlighted several important metabolic pathways, such as the biosynthesis of phenylalanine, tyrosine, and tryptophan, glutathione metabolism, and arginine and proline metabolism. These pathways were enriched not only among the differential metabolites but also within the modules correlated with depressive features. Consistent with our results, dysregulation of these metabolic pathways has been recognized as the potential pathophysiology of MDD (22–24).

In this study, the DNN model was trained with stochastic gradient descent using back-propagation based on a multi-layer feedforward artificial neural network. Using the DNN algorithms, we identified a signature of seven metabolite biomarkers that effectively discriminated MDD patients from HC. In univariate logistic regression analysis, the results suggested that methionine, tyrosine, and arginine might be protective factors, while the other seven hub metabolites might be risk factors. Furthermore, these metabolites were significantly associated with multiple depressive features, highlighting the complex and interconnected nature of depressive symptomatology. For instance, HexCer(d18:1/24:1) and CE(20:4) positively correlated with total depression scores and somatic symptoms. HexCer(d18:1/24:1) also positively correlated with sadness/depressed mood. Moreover, HexCer(d18:1/24:1), SM(OH)C16:1, PC aa C40:6, and CE(20:4) showed strong associations with insomnia or reduced sleep. Notably, methionine, tyrosine, and arginine significantly exhibited consistent negative correlations with several key depressive features, such as depression severity, sadness/depressed mood, and insomnia. Despite the clinical heterogeneity in MDD, these biomarker detections may still benefit individuals with unreported symptoms or diagnostic uncertainty.

Several recent studies have proposed metabolite-based diagnostic models for MDD. Zhou et al. identified inosine as a promising marker (AUC=0.866) for children and adolescents with MDD (8). Liu et al. used a combination of carnitine C10:1, phosphatidylethanolamine-O 36:5, LysoPE 18:1 sn-2, and tryptophan to discriminate adult patients with MDD from HC, with AUC from 0.838 to 0.869 in the validation set (6). Ma et al. integrated a series of clinical features with common metabolic indicators, achieving the diagnostic performance with an AUC of 0.716 via the CATBoost model (25). In comparison, our model achieved a comparable diagnostic performance (AUC = 0.803) by integrating seven targeted biomarkers from distinct metabolic pathways.

Among the biomarkers identified in this study, tyrosine, methionine, and arginine have been implicated in depression. Our findings further support their potential as biomarkers, as these metabolites not only distinguished patients with MDD from HC but also showed significant associations with specific depressive symptoms. Tyrosine is the precursor of neurotransmitters, and its disrupted metabolism has been implicated in the pathogenesis of depression (26). Consistent with previous studies, we observed significantly lower methionine levels in patients with MDD (27); this dysregulation may contribute to the pathophysiology by reducing oxidative stress in the central nervous system (28). Moreover, methionine is involved in the biosynthesis of S-adenosyl-methionine (SAMe), which has been explored as a dietary supplement for treating depression (29). Significantly reduced arginine levels and global arginine bioavailability ratio (GABR) have been associated with MDD (24, 30); the alterations may disrupt nitric oxide (NO) metabolism, consequently exacerbating oxidative stress in the central nervous system.

Four novel metabolite biomarkers are identified in this study, whose roles in the mechanisms of MDD have not been reported: HexCer(d18:1/24:1), SM (OH) C16:1, CE(20:4), and PC aa C40:6. These metabolites belong to hexosylceramides, sphingomyelins, cholesteryl esters, and phosphatidylcholines, respectively. Hexosylceramides, as the derivatives of ceramides, are rarely reported in patients with MDD; however, they are associated with oxidative stress and neurodegenerative diseases (31). Altered sphingomyelin profiles have been reported in individuals with depression (32). Elevated levels of SM C18:1 and SM C18:0 were found to correlate with higher depression scores in patients with coronary artery disease (33). Research suggested that elevated levels of phosphatidylcholines were associated with depression; for instance, multiple ether-phosphatidylcholine (PC ae) were negatively associated with depressive symptoms (34, 35). Only limited evidence has reported that depression may involve alterations in cholesteryl esters (36, 37).

Several limitations exist in this study. First, the relatively small sample size may limit the robustness of our diagnostic models, necessitating external validation to support them. Second, the study did not account for medication history or genetic background. Third, since metabolomics is not routinely tested in clinical practice for MDD, the high costs may hinder the clinical translation of these metabolites. To promote the incorporation of these findings as a fast and reliable diagnostic tool, further studies with larger sample sizes across diverse regions and ethnicities are needed to independently validate the clinical application of the biomarkers identified in this research. Additionally, we should explore alternative detection methods for the identified metabolites, such as simplified mass spectrometry techniques and new detection strategies (e.g., assay kits), to facilitate their clinical applications.

## Conclusion

This diagnostic study identified hub metabolites and significant pathways associated with depressive features through the metabolite co-expression network. Based on a metabolomic biomarker signature, we developed a highly interpretable diagnostic model that effectively discriminated between the MDD and HC groups. These findings provide new insights into the biological diagnosis of MDD and provide potential pathways for intervention.

## Data Availability

The raw data supporting the conclusions of this article will be made available by the authors, without undue reservation.
