# Supplementary material for: A network-based approach to discover diagnostic metabolite markers associated with depressive features for major depressive disorder
Source: Front Psychiatry. 2025 Jun 6;16:1610520. doi: 10.3389/fpsyt.2025.1610520 (PMC12179064; doi:10.3389/fpsyt.2025.1610520)
Supplement: Supplementary file 1 [file DataSheet1.zip › Supplementary Tables.docx]

**Supplementary Table S1. List of metabolites measured by metabolomics**

| **Class** | **Number** | **Name** | **Full name** |
| --- | --- | --- | --- |
| Alkaloids | 1 | Trigonelline | Trigonelline |
| Amine Oxides | 1 | TMAO | Trimethylamine N-oxide |
| Amino Acids | 20 | Ala | Alanine |
|  |  | Arg | Arginine |
|  |  | Asn | Asparagine |
|  |  | Asp | Aspartate |
|  |  | Cys | Cysteine |
|  |  | Glu | Glutamate |
|  |  | Gln | Glutamine |
|  |  | Gly | Glycine |
|  |  | His | Histidine |
|  |  | Ile | Isoleucine |
|  |  | Leu | Leucine |
|  |  | Lys | Lysine |
|  |  | Met | Methionine |
|  |  | Phe | Phenylalanine |
|  |  | Pro | Proline |
|  |  | Ser | Serine |
|  |  | Thr | Threonine |
|  |  | Trp | Tryptophan |
|  |  | Tyr | Tyrosine |
|  |  | Val | Valine |
| Amino Acid Related | 30 | alpha-AAA | α-Aminoadipic acid |
|  |  | AABA | α -Aminobutyric acid |
|  |  | Ac-Orn | Acetylornithine |
|  |  | ADMA | Asymmetric dimethylarginine |
|  |  | Anserine | Anserine |
|  |  | 5-AVA | 5-Aminovaleric acid |
|  |  | BABA | β-Aminobutyric acid |
|  |  | Betaine | Betaine |
|  |  | Carnosine | Carnosine |
|  |  | Cit | Citrulline |
|  |  | Creatinine | Creatinine |
|  |  | Cystine | Cystine |
|  |  | DOPA | Dihydroxyphenylalanine |
|  |  | HArg | Homoarginine |
|  |  | HCys | Homocysteine |
|  |  | c4-OH-Pro | cis-4-Hydroxyproline |
|  |  | t4-OH-Pro | trans-4-Hydroxyproline |
|  |  | Kynurenine | Kynurenine |
|  |  | Met-SO | Methionine sulfoxide |
|  |  | 1-Met-His | 1-Methylhistidine |
|  |  | 3-Met-His | 3-Methylhistidine |
|  |  | Nitro-Tyr | Nitrotyrosine |
|  |  | Orn | Ornithine |
|  |  | PAG | Phenylacetylglycine |
|  |  | PheAlaBetaine | Phenylalanine betaine |
|  |  | ProBetaine | Proline betaine |
|  |  | Sarcosine | Sarcosine |
|  |  | SDMA | Symmetric dimethylarginine |
|  |  | Taurine | Taurine |
|  |  | TrpBetaine | Tryptophan betaine |
| Bile Acids | 14 | CA | Cholic acid |
|  |  | CDCA | Chenodeoxycholic acid |
|  |  | DCA | Deoxycholic acid |
|  |  | GCA | Glycocholic acid |
|  |  | GDCA | Glycodeoxycholic acid |
|  |  | GCDCA | Glycochenodeoxycholic acid |
|  |  | GLCA | Glycolithocholic acid |
|  |  | GLCAS | Glycolithocholic acid sulfate |
|  |  | GUDCA | Glycoursodeoxycholic acid |
|  |  | TCA | Taurocholic acid |
|  |  | TCDCA | Taurochenodeoxycholic acid |
|  |  | TDCA | Taurodeoxycholic acid |
|  |  | TLCA | Taurolithocholic acid |
|  |  | TMCA | Tauromurocholic acid |
| Biogenic Amines | 9 | beta-Ala | β-Alanine |
|  |  | GABA | γ-Aminobutyric acid |
|  |  | Dopamine | Dopamine |
|  |  | Histamine | Histamine |
|  |  | PEA | Phenylethylamine |
|  |  | Putrescine | Putrescine |
|  |  | Serotonin | Serotonin |
|  |  | Spermidine | Spermidine |
|  |  | Spermine | Spermine |
| Carbohydrates and Related | 1 | H1 | Hexoses (including glucose) |
| Carboxylic Acids | 7 | AconAcid | Aconitic acid |
|  |  | DiCA(12:0) | Dodecanedioic acid |
|  |  | DiCA(14:0) | Tetradecanedioic acid |
|  |  | HipAcid | Hippuric acid |
|  |  | OH-GlutAcid | 3-Hydroxyglutaric acid |
|  |  | Lac | Lactic acid |
|  |  | Suc | Succinic acid |
| Cresols | 1 | p-Cresol-SO4 | p-Cresol sulfate |
|  |  | FA(12:0) | Lauric acid |
|  |  | FA(14:0) | Myristic acid |
|  |  | FA(16:0) | Palmitic acid |
|  |  | FA(18:0) | Stearic acid |
| Fatty Acids | 12 | FA(18:1) | Octadecenoic acid |
|  |  | FA(18:2) | Octadecadienoic acid |
|  |  | FA(20:1) | Eicosenoic acid |
|  |  | FA(20:2) | Eicosadienoic acid |
|  |  | FA(20:3) | Eicosatrienoic acid |
|  |  | AA | Arachidonic acid (FA(20:4ω6)) |
|  |  | EPA | Eicosapentaenoic acid (FA(20:5ω3)) |
|  |  | DHA | Docosahexaenoid acid (FA(22:6ω3)) |
| Hormones and Related | 4 | AbsAcid | Abscisic acid |
|  |  | Cortisol | Cortisol |
|  |  | Cortisone | Cortisone |
|  |  | DHEAS | Dehydroepiandrosterone sulfate |
| Indoles and Derivatives | 4 | Indole | Indole |
|  |  | 3-IAA | 3-Indoleacetic acid |
|  |  | 3-IPA | 3-Indolepropionic acid |
|  |  | Ind-SO4 | Indoxyl sulfate |
| Nucleobases and Related | 2 | Hypoxanthine | Hypoxanthine |
|  |  | Xanthine | Xanthine |
| Vitamins and Cofactors | 1 | Choline | Choline |
| Acylcarnitines | 40 | C0 | Carnitine |
|  |  | C2 | Acetylcarnitine |
|  |  | C3 | Propionylcarnitine |
|  |  | C3-DC(C4-OH) | Malonylcarnitine (Hydroxybutyrylcarnitine) |
|  |  | C3-OH | Hydroxypropionylcarnitine |
|  |  | C3:1 | Propenoylcarnitine |
|  |  | C4 | Butyrylcarnitine |
|  |  | C4:1 | Butenylcarnitine |
|  |  | C5 | Valerylcarnitine |
|  |  | C5-DC(C6-OH) | Glutarylcarnitine (Hydroxyhexanoylcarnitine) |
|  |  | C5-M-DC | Methylglutarylcarnitine |
|  |  | C5-OH(C3-DC-M) | Hydroxyvalerylcarnitine (Methylmalonylcarnitine) |
|  |  | C5:1 | Tiglylcarnitine |
|  |  | C5:1-DC | Glutaconylcarnitine |
|  |  | C6 (C4:1-DC) | Hexanoylcarnitine (Fumarylcarnitine) |
|  |  | C6:1 | Hexenoylcarnitine |
|  |  | C7-DC | Pimeloylcarnitine |
|  |  | C8 | Octanoylcarnitine |
|  |  | C9 | Nonaylcarnitine |
|  |  | C10 | Decanoylcarnitine |
|  |  | C10:1 | Decenoylcarnitine |
|  |  | C10:2 | Decadienoylcarnitine |
|  |  | C12 | Dodecanoylcarnitine |
|  |  | C12-DC | Dodecanedioylcarnitine |
|  |  | C12:1 | Dodecenoylcarnitine |
|  |  | C14 | Tetradecanoylcarnitine |
|  |  | C14:1 | Tetradecenoylcarnitine |
|  |  | C14:1-OH | Hydroxytetradecenoylcarnitine |
|  |  | C14:2 | Tetradecadienoylcarnitine |
|  |  | C14:2-OH | Hydroxytetradecadienoylcarnitine |
|  |  | C16 | Hexadecanoylcarnitine |
|  |  | C16-OH | Hydroxyhexadecanoylcarnitine |
|  |  | C16:1 | Hexadecenoylcarnitine |
|  |  | C16:1-OH | Hydroxyhexadecenoylcarnitine |
|  |  | C16:2 | Hexadecadienoylcarnitine |
|  |  | C16:2-OH | Hydroxyhexadecadienoylcarnitine |
|  |  | C18 | Octadecanoylcarnitine |
|  |  | C18:1 | Octadecenoylcarnitine |
|  |  | C18:1-OH | Hydroxyoctadecenoylcarnitine |
|  |  | C18:2 | Octadecadienylcarnitine |
| Lysophosphatidylcholines | 14 | lysoPC a C14:0 |  |
|  |  | lysoPC a C16:0 |  |
|  |  | lysoPC a C16:1 |  |
|  |  | lysoPC a C17:0 |  |
|  |  | lysoPC a C18:0 |  |
|  |  | lysoPC a C18:1 |  |
|  |  | lysoPC a C18:2 |  |
|  |  | lysoPC a C20:3 |  |
|  |  | lysoPC a C20:4 |  |
|  |  | lysoPC a C24:0 |  |
|  |  | lysoPC a C26:0 |  |
|  |  | lysoPC a C26:1 |  |
|  |  | lysoPC a C28:0 |  |
|  |  | lysoPC a C28:1 |  |
| Phosphatidylcholines | 76 | PC aa C24:0 |  |
|  |  | PC aa C26:0 |  |
|  |  | PC aa C28:1 |  |
|  |  | PC aa C30:0 |  |
|  |  | PC aa C30:2 |  |
|  |  | PC aa C32:0 |  |
|  |  | PC aa C32:1 |  |
|  |  | PC aa C32:2 |  |
|  |  | PC aa C32:3 |  |
|  |  | PC aa C34:1 |  |
|  |  | PC aa C34:2 |  |
|  |  | PC O-16:1_19:1 |  |
|  |  | PC aa C34:4 |  |
|  |  | PC aa C36:0 |  |
|  |  | PC aa C36:1 |  |
|  |  | PC aa C36:2 |  |
|  |  | PC aa C36:3 |  |
|  |  | PC aa C36:4 |  |
|  |  | PC 18:2_18:2 |  |
|  |  | PC aa C36:6 |  |
|  |  | PC aa C38:0 |  |
|  |  | PC aa C38:1 |  |
|  |  | PC O-18:1_21:0 |  |
|  |  | PC aa C38:4 |  |
|  |  | PC aa C38:5 |  |
|  |  | PC aa C38:6 |  |
|  |  | PC aa C40:1 |  |
|  |  | PC aa C40:2 |  |
|  |  | PC 20:1_20:1 |  |
|  |  | PC 18:3_22:0 |  |
|  |  | PC aa C40:5 |  |
|  |  | PC aa C40:6 |  |
|  |  | PC aa C42:0 |  |
|  |  | PC 20:0_22:0 |  |
|  |  | PC 20:1_22:0 |  |
|  |  | PC aa C42:4 |  |
|  |  | PC aa C42:5 |  |
|  |  | PC aa C42:6 |  |
|  |  | PC ae C30:0 |  |
|  |  | PC ae C30:1 |  |
|  |  | PC ae C30:2 |  |
|  |  | PC ae C32:1 |  |
|  |  | PC ae C32:2 |  |
|  |  | PC ae C34:0 |  |
|  |  | PC ae C34:1 |  |
|  |  | PC ae C34:2 |  |
|  |  | PC ae C34:3 |  |
|  |  | PC ae C36:0 |  |
|  |  | PC ae C36:1 |  |
|  |  | PC ae C36:2 |  |
|  |  | PC ae C36:3 |  |
|  |  | PC 17:2_18:1 |  |
|  |  | PC ae C36:5 |  |
|  |  | PC ae C38:0 |  |
|  |  | PC ae C38:1 |  |
|  |  | PC ae C38:2 |  |
|  |  | PC ae C38:3 |  |
|  |  | PC ae C38:4 |  |
|  |  | PC ae C38:5 |  |
|  |  | PC ae C38:6 |  |
|  |  | PC aa C40:1 |  |
|  |  | PC ae C40:2 |  |
|  |  | PC ae C40:3 |  |
|  |  | PC ae C40:4 |  |
|  |  | PC ae C40:5 |  |
|  |  | PC ae C40:6 |  |
|  |  | PC ae C42:0 |  |
|  |  | PC ae C42:1 |  |
|  |  | PC ae C42:2 |  |
|  |  | PC ae C42:3 |  |
|  |  | PC ae C42:4 |  |
|  |  | PC ae C42:5 |  |
|  |  | PC ae C44:3 |  |
|  |  | PC ae C44:4 |  |
|  |  | PC ae C44:5 |  |
|  |  | PC ae C44:6 |  |
| Sphingomyelins | 15 | SM C16:0 |  |
|  |  | SM C16:1 |  |
|  |  | SM d16:1/18:1 |  |
|  |  | SM d18:1/18:0 |  |
|  |  | SM d18:1/18:1 |  |
|  |  | SM d18:1/20:2 |  |
|  |  | SM C24:0 |  |
|  |  | SM d18:1/24:0 |  |
|  |  | SM d18:1/24:0 |  |
|  |  | SM C26:1 |  |
|  |  | SM (OH) C14:1 |  |
|  |  | SM d18:1/15:0 |  |
|  |  | SM (OH) C22:1 |  |
|  |  | SM d16:1/25:0 |  |
|  |  | SM (OH) C24:1 |  |
| Ceramides | 28 | Cer(d16:1/18:0) |  |
|  |  | Cer(d16:1/20:0) |  |
|  |  | Cer(d16:1/22:0) |  |
|  |  | Cer(d16:1/23:0) |  |
|  |  | Cer(d16:1/24:0) |  |
|  |  | Cer(d18:1/14:0) |  |
|  |  | Cer(d18:1/16:0) |  |
|  |  | Cer(d18:1/18:0(OH)) |  |
|  |  | Cer(d18:1/18:0) |  |
|  |  | Cer(d18:1/18:1) |  |
|  |  | Cer(d18:1/20:0(OH)) |  |
|  |  | Cer(d18:1/20:0) |  |
|  |  | Cer(d18:1/22:0) |  |
|  |  | Cer(d18:1/23:0) |  |
|  |  | Cer(d18:1/24:0) |  |
|  |  | Cer(d18:1/24:1) |  |
|  |  | Cer(d18:1/25:0) |  |
|  |  | Cer(d18:1/26:0) |  |
|  |  | Cer(d18:1/26:1) |  |
|  |  | Cer(d18:2/14:0) |  |
|  |  | Cer(d18:2/16:0) |  |
|  |  | Cer(d18:2/18:0) |  |
|  |  | Cer(d18:2/18:1) |  |
|  |  | Cer(d18:2/20:0) |  |
|  |  | Cer(d18:2/22:0) |  |
|  |  | Cer(d18:2/23:0) |  |
|  |  | Cer(d18:2/24:0) |  |
|  |  | Cer(d18:2/24:1) |  |
| Dihydroceramides | 8 | Cer(d18:0/18:0(OH)) |  |
|  |  | Cer(d18:0/18:0) |  |
|  |  | Cer(d18:0/20:0) |  |
|  |  | Cer(d18:0/22:0) |  |
|  |  | Cer(d18:0/24:0) |  |
|  |  | Cer(d18:0/24:1) |  |
|  |  | Cer(d18:0/26:1(OH)) |  |
|  |  | Cer(d18:0/26:1) |  |
| Hexosylceramides | 19 | HexCer(d16:1/22:0) |  |
|  |  | HexCer(d16:1/24:0) |  |
|  |  | HexCer(d18:1/14:0) |  |
|  |  | HexCer(d18:1/16:0) |  |
|  |  | HexCer(d18:1/18:0) |  |
|  |  | HexCer(d18:1/18:1) |  |
|  |  | HexCer(d18:1/20:0) |  |
|  |  | HexCer(d18:1/22:0) |  |
|  |  | HexCer(d18:1/23:0) |  |
|  |  | HexCer(d18:1/24:0) |  |
|  |  | HexCer(d18:1/24:1) |  |
|  |  | HexCer(d18:1/26:0) |  |
|  |  | HexCer(d18:1/26:1) |  |
|  |  | HexCer(d18:2/16:0) |  |
|  |  | HexCer(d18:2/18:0) |  |
|  |  | HexCer(d18:2/20:0) |  |
|  |  | HexCer(d18:2/22:0) |  |
|  |  | HexCer(d18:2/23:0) |  |
|  |  | HexCer(d18:2/24:0) |  |
| Dihexosylceramides | 9 | Hex2Cer(d18:1/14:0) |  |
|  |  | Hex2Cer(d18:1/16:0) |  |
|  |  | Hex2Cer(d18:1/18:0) |  |
|  |  | Hex2Cer(d18:1/20:0) |  |
|  |  | Hex2Cer(d18:1/22:0) |  |
|  |  | Hex2Cer(d18:1/24:0) |  |
|  |  | Hex2Cer(d18:1/24:1) |  |
|  |  | Hex2Cer(d18:1/26:0) |  |
|  |  | Hex2Cer(d18:1/26:1) |  |
| Trihexosylceramides | 6 | Hex3Cer(d18:1/16:0) |  |
|  |  | Hex3Cer(d18:1/18:0) |  |
|  |  | Hex3Cer(d18:1_20:0) |  |
|  |  | Hex3Cer(d18:1_22:0) |  |
|  |  | Hex3Cer(d18:1/24:1) |  |
|  |  | Hex3Cer(d18:1/26:1) |  |
| Cholesteryl esters | 22 | CE(14:0) |  |
|  |  | CE(14:1) |  |
|  |  | CE(15:0) |  |
|  |  | CE(15:1) |  |
|  |  | CE(16:0) |  |
|  |  | CE(16:1) |  |
|  |  | CE(17:0) |  |
|  |  | CE(17:1) |  |
|  |  | CE(18:0) |  |
|  |  | CE(18:1) |  |
|  |  | CE(18:2) |  |
|  |  | CE(18:3) |  |
|  |  | CE(20:0) |  |
|  |  | CE(20:1) |  |
|  |  | CE(20:3) |  |
|  |  | CE(20:4) |  |
|  |  | CE(20:5) |  |
|  |  | CE(22:0) |  |
|  |  | CE(22:1) |  |
|  |  | CE(22:2) |  |
|  |  | CE(22:5) |  |
|  |  | CE(22:6) |  |
| Diglycerides | 44 | DG(14:0_14:0) |  |
|  |  | DG(14:0_18:1) |  |
|  |  | DG(14:0_18:2) |  |
|  |  | DG(14:0_20:0) |  |
|  |  | DG(14:1_18:1) |  |
|  |  | DG(14:1_20:2) |  |
|  |  | DG(16:0_16:0) |  |
|  |  | DG(16:0_16:1) |  |
|  |  | DG(16:0_18:1) |  |
|  |  | DG(16:0_18:2) |  |
|  |  | DG(16:0_20:0) |  |
|  |  | DG(16:0_20:3) |  |
|  |  | DG(16:0_20:4) |  |
|  |  | DG(16:1_18:0) |  |
|  |  | DG(16:1_18:1) |  |
|  |  | DG(16:1_18:2) |  |
|  |  | DG(16:1_20:0) |  |
|  |  | DG(17:0_17:1) |  |
|  |  | DG(17:0_18:1) |  |
|  |  | DG(18:0_20:4) |  |
|  |  | DG(18:0_22:6) |  |
|  |  | DG(18:1_18:1) |  |
|  |  | DG(18:1_18:2) |  |
|  |  | DG(18:1_18:3) |  |
|  |  | DG(18:1_18:4) |  |
|  |  | DG(18:1_20:0) |  |
|  |  | DG(18:1_20:1) |  |
|  |  | DG(18:1_20:2) |  |
|  |  | DG(18:1_20:3) |  |
|  |  | DG(18:1_20:4) |  |
|  |  | DG(18:1_22:5) |  |
|  |  | DG(18:1_22:6) |  |
|  |  | DG(18:2_18:2) |  |
|  |  | DG(18:2_18:3) |  |
|  |  | DG(18:2_18:4) |  |
|  |  | DG(18:2_20:0) |  |
|  |  | DG(18:2_20:4) |  |
|  |  | DG(18:3_18:3) |  |
|  |  | DG(18:3_20:2) |  |
|  |  | DG(21:0_22:6) |  |
|  |  | DG(22:1_22:2) |  |
|  |  | DG-O(14:0_18:2) |  |
|  |  | DG-O(16:0_18:1) |  |
|  |  | DG-O(16:0_20:4) |  |
| Triglycerides | 242 | TG(14:0_32:2) |  |
|  |  | TG(14:0_34:0) |  |
|  |  | TG(14:0_34:1) |  |
|  |  | TG(14:0_34:2) |  |
|  |  | TG(14:0_34:3) |  |
|  |  | TG(14:0_35:1) |  |
|  |  | TG(14:0_35:2) |  |
|  |  | TG(14:0_36:1) |  |
|  |  | TG(14:0_36:2) |  |
|  |  | TG(14:0_36:3) |  |
|  |  | TG(14:0_36:4) |  |
|  |  | TG(14:0_38:4) |  |
|  |  | TG(14:0_38:5) |  |
|  |  | TG(14:0_39:3) |  |
|  |  | TG(16:0_28:1) |  |
|  |  | TG(16:0_28:2) |  |
|  |  | TG(16:0_30:2) |  |
|  |  | TG(16:0_32:0) |  |
|  |  | TG(16:0_32:1) |  |
|  |  | TG(16:0_32:2) |  |
|  |  | TG(16:0_32:3) |  |
|  |  | TG(16:0_33:1) |  |
|  |  | TG(16:0_33:2) |  |
|  |  | TG(16:0_34:0) |  |
|  |  | TG(16:0_34:1) |  |
|  |  | TG(16:0_34:2) |  |
|  |  | TG(16:0_34:3) |  |
|  |  | TG(16:0_34:4) |  |
|  |  | TG(16:0_35:1) |  |
|  |  | TG(16:0_35:2) |  |
|  |  | TG(16:0_35:3) |  |
|  |  | TG(16:0_36:2) |  |
|  |  | TG(16:0_36:3) |  |
|  |  | TG(16:0_36:4) |  |
|  |  | TG(16:0_36:5) |  |
|  |  | TG(16:0_36:6) |  |
|  |  | TG(16:0_37:3) |  |
|  |  | TG(16:0_38:1) |  |
|  |  | TG(16:0_38:2) |  |
|  |  | TG(16:0_38:3) |  |
|  |  | TG(16:0_38:4) |  |
|  |  | TG(16:0_38:5) |  |
|  |  | TG(16:0_38:6) |  |
|  |  | TG(16:0_38:7) |  |
|  |  | TG(16:0_40:6) |  |
|  |  | TG(16:0_40:7) |  |
|  |  | TG(16:0_40:8) |  |
|  |  | TG(16:1_28:0) |  |
|  |  | TG(16:1_30:1) |  |
|  |  | TG(16:1_32:0) |  |
|  |  | TG(16:1_32:1) |  |
|  |  | TG(16:1_32:2) |  |
|  |  | TG(16:1_33:1) |  |
|  |  | TG(16:1_34:0) |  |
|  |  | TG(16:1_34:1) |  |
|  |  | TG(16:1_34:2) |  |
|  |  | TG(16:1_34:3) |  |
|  |  | TG(16:1_36:1) |  |
|  |  | TG(16:1_36:2) |  |
|  |  | TG(16:1_36:3) |  |
|  |  | TG(16:1_36:4) |  |
|  |  | TG(16:1_36:5) |  |
|  |  | TG(16:1_38:3) |  |
|  |  | TG(16:1_38:4) |  |
|  |  | TG(16:1_38:5) |  |
|  |  | TG(17:0_32:1) |  |
|  |  | TG(17:0_34:1) |  |
|  |  | TG(17:0_34:2) |  |
|  |  | TG(17:0_34:3) |  |
|  |  | TG(17:0_36:3) |  |
|  |  | TG(17:0_36:4) |  |
|  |  | TG(17:1_32:1) |  |
|  |  | TG(17:1_34:1) |  |
|  |  | TG(17:1_34:2) |  |
|  |  | TG(17:1_34:3) |  |
|  |  | TG(17:1_36:3) |  |
|  |  | TG(17:1_36:4) |  |
|  |  | TG(17:1_36:5) |  |
|  |  | TG(17:1_38:5) |  |
|  |  | TG(17:1_38:6) |  |
|  |  | TG(17:1_38:7) |  |
|  |  | TG(17:2_34:2) |  |
|  |  | TG(17:2_34:3) |  |
|  |  | TG(17:2_36:2) |  |
|  |  | TG(17:2_36:3) |  |
|  |  | TG(17:2_36:4) |  |
|  |  | TG(17:2_38:5) |  |
|  |  | TG(17:2_38:6) |  |
|  |  | TG(17:2_38:7) |  |
|  |  | TG(18:0_30:0) |  |
|  |  | TG(18:0_30:1) |  |
|  |  | TG(18:0_32:0) |  |
|  |  | TG(18:0_32:1) |  |
|  |  | TG(18:0_32:2) |  |
|  |  | TG(18:0_34:2) |  |
|  |  | TG(18:0_34:3) |  |
|  |  | TG(18:0_36:1) |  |
|  |  | TG(18:0_36:2) |  |
|  |  | TG(18:0_36:3) |  |
|  |  | TG(18:0_36:4) |  |
|  |  | TG(18:0_36:5) |  |
|  |  | TG(18:0_38:6) |  |
|  |  | TG(18:0_38:7) |  |
|  |  | TG(18:1_26:0) |  |
|  |  | TG(18:1_28:1) |  |
|  |  | TG(18:1_30:0) |  |
|  |  | TG(18:1_30:1) |  |
|  |  | TG(18:1_30:2) |  |
|  |  | TG(18:1_31:0) |  |
|  |  | TG(18:1_32:0) |  |
|  |  | TG(18:1_32:1) |  |
|  |  | TG(18:1_32:2) |  |
|  |  | TG(18:1_32:3) |  |
|  |  | TG(18:1_33:0) |  |
|  |  | TG(18:1_33:1) |  |
|  |  | TG(18:1_33:2) |  |
|  |  | TG(18:1_33:3) |  |
|  |  | TG(18:1_34:1) |  |
|  |  | TG(18:1_34:2) |  |
|  |  | TG(18:1_34:3) |  |
|  |  | TG(18:1_34:4) |  |
|  |  | TG(18:1_35:2) |  |
|  |  | TG(18:1_35:3) |  |
|  |  | TG(18:1_36:0) |  |
|  |  | TG(18:1_36:1) |  |
|  |  | TG(18:1_36:2) |  |
|  |  | TG(18:1_36:3) |  |
|  |  | TG(18:1_36:4) |  |
|  |  | TG(18:1_36:5) |  |
|  |  | TG(18:1_36:6) |  |
|  |  | TG(18:1_38:5) |  |
|  |  | TG(18:1_38:6) |  |
|  |  | TG(18:1_38:7) |  |
|  |  | TG(18:2_28:0) |  |
|  |  | TG(18:2_30:0) |  |
|  |  | TG(18:2_30:1) |  |
|  |  | TG(18:2_31:0) |  |
|  |  | TG(18:2_32:0) |  |
|  |  | TG(18:2_32:1) |  |
|  |  | TG(18:2_32:2) |  |
|  |  | TG(18:2_33:0) |  |
|  |  | TG(18:2_33:1) |  |
|  |  | TG(18:2_33:2) |  |
|  |  | TG(18:2_34:0) |  |
|  |  | TG(18:2_34:1) |  |
|  |  | TG(18:2_34:2) |  |
|  |  | TG(18:2_34:3) |  |
|  |  | TG(18:2_34:4) |  |
|  |  | TG(18:2_35:1) |  |
|  |  | TG(18:2_35:2) |  |
|  |  | TG(18:2_35:3) |  |
|  |  | TG(18:2_36:0) |  |
|  |  | TG(18:2_36:1) |  |
|  |  | TG(18:2_36:2) |  |
|  |  | TG(18:2_36:3) |  |
|  |  | TG(18:2_36:4) |  |
|  |  | TG(18:2_36:5) |  |
|  |  | TG(18:2_38:4) |  |
|  |  | TG(18:2_38:5) |  |
|  |  | TG(18:2_38:6) |  |
|  |  | TG(18:3_30:0) |  |
|  |  | TG(18:3_32:0) |  |
|  |  | TG(18:3_32:1) |  |
|  |  | TG(18:3_33:2) |  |
|  |  | TG(18:3_34:0) |  |
|  |  | TG(18:3_34:1) |  |
|  |  | TG(18:3_34:2) |  |
|  |  | TG(18:3_34:3) |  |
|  |  | TG(18:3_35:2) |  |
|  |  | TG(18:3_36:1) |  |
|  |  | TG(18:3_36:2) |  |
|  |  | TG(18:3_36:3) |  |
|  |  | TG(18:3_36:4) |  |
|  |  | TG(18:3_38:5) |  |
|  |  | TG(18:3_38:6) |  |
|  |  | TG(20:0_32:3) |  |
|  |  | TG(20:0_32:4) |  |
|  |  | TG(20:0_34:1) |  |
|  |  | TG(20:1_24:3) |  |
|  |  | TG(20:1_26:1) |  |
|  |  | TG(20:1_30:1) |  |
|  |  | TG(20:1_31:0) |  |
|  |  | TG(20:1_32:1) |  |
|  |  | TG(20:1_32:2) |  |
|  |  | TG(20:1_32:3) |  |
|  |  | TG(20:1_34:0) |  |
|  |  | TG(20:1_34:1) |  |
|  |  | TG(20:1_34:2) |  |
|  |  | TG(20:1_34:3) |  |
|  |  | TG(20:2_32:0) |  |
|  |  | TG(20:2_32:1) |  |
|  |  | TG(20:2_34:1) |  |
|  |  | TG(20:2_34:2) |  |
|  |  | TG(20:2_34:3) |  |
|  |  | TG(20:2_34:4) |  |
|  |  | TG(20:2_36:5) |  |
|  |  | TG(20:3_32:0) |  |
|  |  | TG(20:3_32:1) |  |
|  |  | TG(20:3_32:2) |  |
|  |  | TG(20:3_34:0) |  |
|  |  | TG(20:3_34:1) |  |
|  |  | TG(20:3_34:2) |  |
|  |  | TG(20:3_34:3) |  |
|  |  | TG(20:3_36:3) |  |
|  |  | TG(20:3_36:4) |  |
|  |  | TG(20:3_36:5) |  |
|  |  | TG(20:4_30:0) |  |
|  |  | TG(20:4_32:0) |  |
|  |  | TG(20:4_32:1) |  |
|  |  | TG(20:4_32:2) |  |
|  |  | TG(20:4_33:2) |  |
|  |  | TG(20:4_34:0) |  |
|  |  | TG(20:4_34:1) |  |
|  |  | TG(20:4_34:2) |  |
|  |  | TG(20:4_34:2) |  |
|  |  | TG(20:4_35:3) |  |
|  |  | TG(20:4_36:2) |  |
|  |  | TG(20:4_36:3) |  |
|  |  | TG(20:4_36:4) |  |
|  |  | TG(20:4_36:5) |  |
|  |  | TG(20:5_34:0) |  |
|  |  | TG(20:5_34:1) |  |
|  |  | TG(20:5_34:2) |  |
|  |  | TG(20:5_36:2) |  |
|  |  | TG(20:5_36:3) |  |
|  |  | TG(22:0_32:4) |  |
|  |  | TG(22:1_32:5) |  |
|  |  | TG(22:2_32:4) |  |
|  |  | TG(22:3_30:2) |  |
|  |  | TG(22:4_32:0) |  |
|  |  | TG(22:4_32:2) |  |
|  |  | TG(22:4_34:2) |  |
|  |  | TG(22:5_32:0) |  |
|  |  | TG(22:5_32:1) |  |
|  |  | TG(22:5_34:1) |  |
|  |  | TG(22:5_34:2) |  |
|  |  | TG(22:5_34:3) |  |
|  |  | TG(22:6_32:0) |  |
|  |  | TG(22:6_32:1) |  |
|  |  | TG(22:6_34:1) |  |
|  |  | TG(22:6_34:2) |  |
|  |  | TG(22:6_34:3) |  |

**Supplementary Table S2. Parameters and R packages for machine learning**

| **Machine learning** | **Parameters** | **R packages** |
| --- | --- | --- |
| **Feature selection  （5-fold cross-validation）** |  |  |
| Elastic net | alpha = 0.9, lambda=0.02853 | *glmnet* |
| **Model training  (5-fold cross-validation)** |  |  |
| Ridge regression | lambda=0.204 | *glmnet* |
| Naive Bayes | laplace = 0, usekernel = F, adjust = 1, kernel = "gaussian" , prior = c(0.33, 0.67) | *naivebayes* |
| Random forest | ntree =25, mtry=2, nodesize=18 | *randomForest* |
| Support vector machine | kernel="radial", gamma=0.01, cost=0.2 | *e1071* |
| XGBoost | booster = "gbtree", eval_metric = "error",  eta = 0.01, max_depth = 2,  gamma = 1, colsample_bytree = 0.4,  min_child_weight = 3, subsample = 0.5 | *xgboost* |
| DNN | layer units (7-15-15-2)，  Rectifier Dropout 10.0 % | *h2o* |
