# Supplementary material for: A network-based approach to discover diagnostic metabolite markers associated with depressive features for major depressive disorder
Source: Front Psychiatry. 2025 Jun 6;16:1610520. doi: 10.3389/fpsyt.2025.1610520 (PMC12179064; doi:10.3389/fpsyt.2025.1610520)
Supplement: Supplementary file 1 [file DataSheet1.zip › Supplementary Figures.docx]

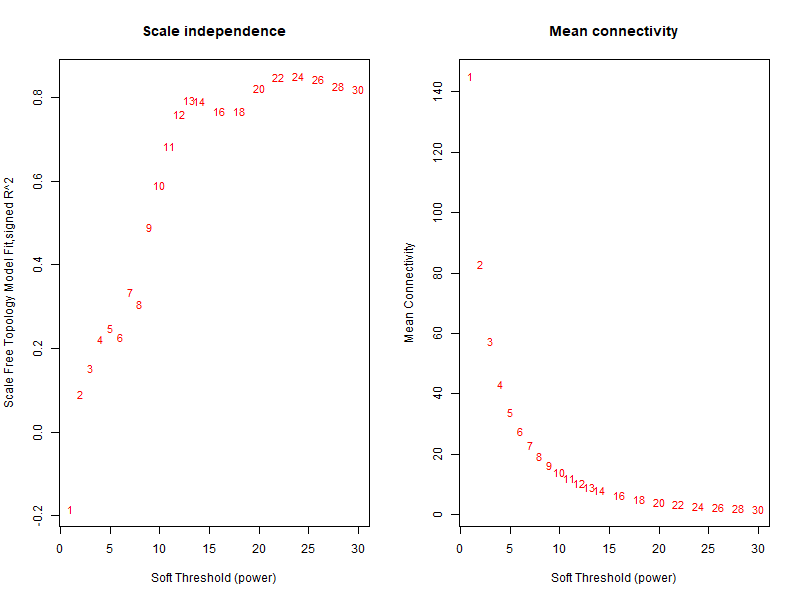


**Supplementary Figure S1. Scale independence and mean connectivity with different soft threshold power**


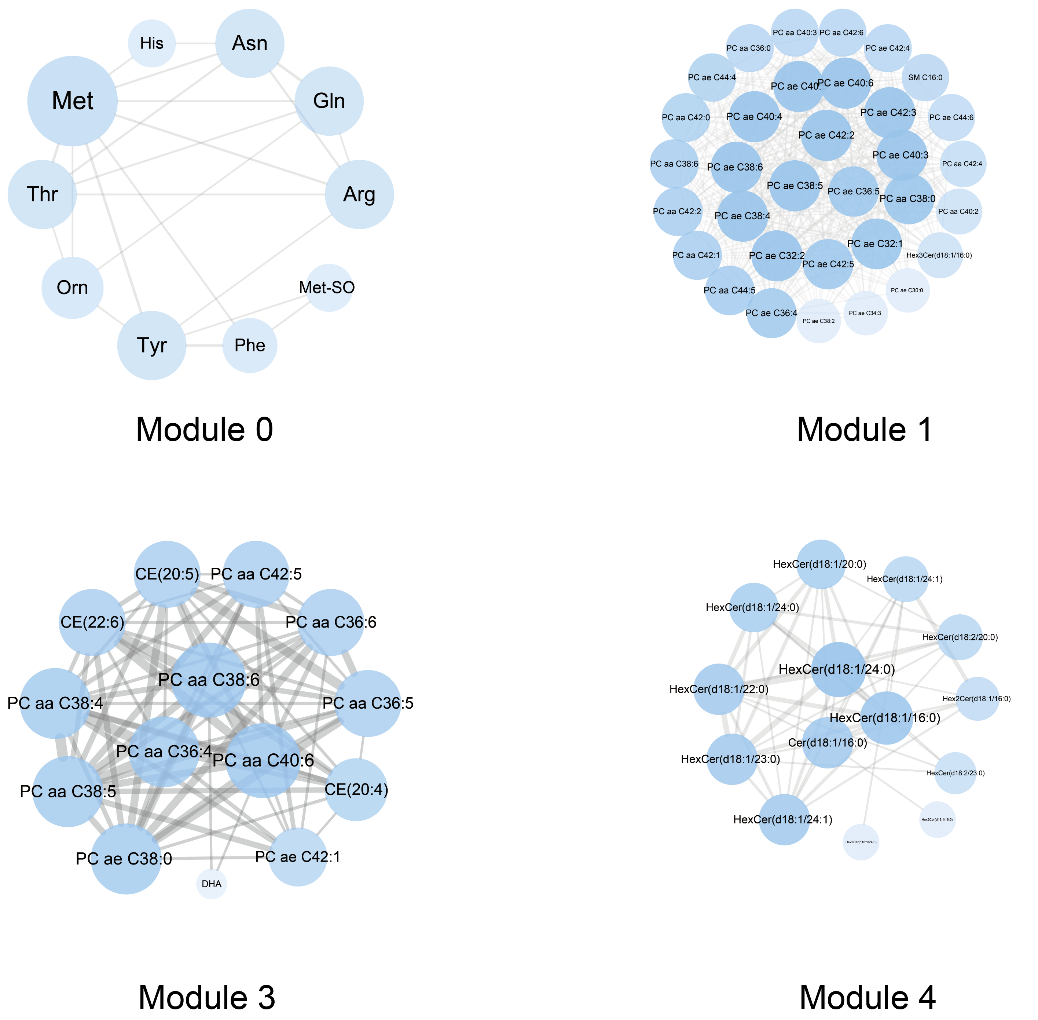


**Supplementary Figure S2. Primary interaction network of metabolites within the four significant modules.**


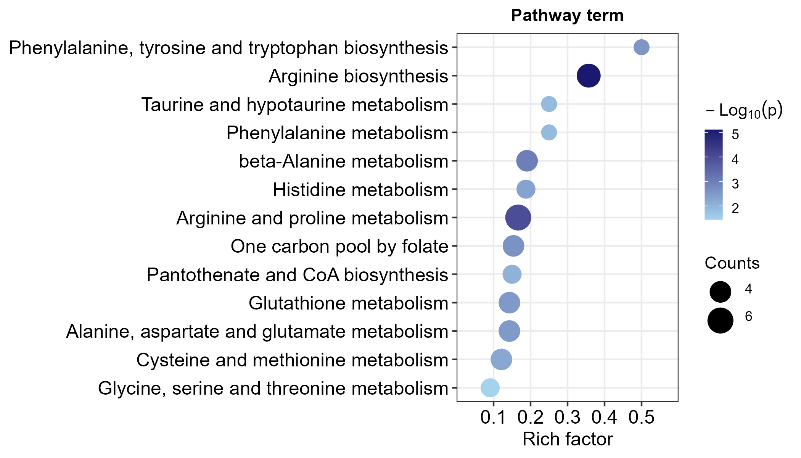


**Supplementary Figure S3. Metabolic pathway enrichment analysis of metabolites in the four significant modules**


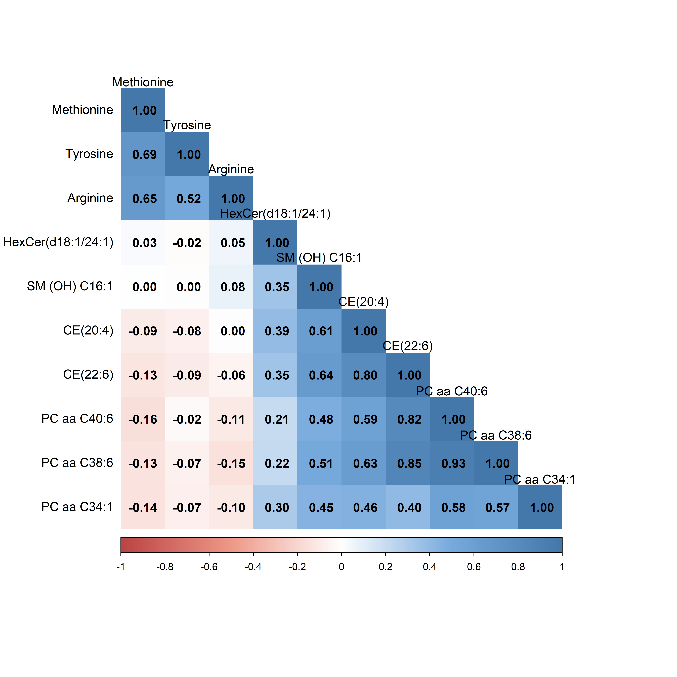


**Supplementary Figure S4.** **Correlation analysis among the ten hub metabolites**


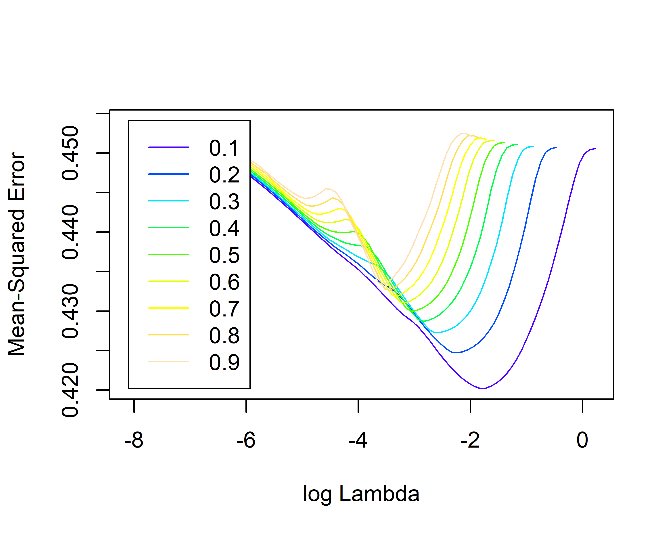

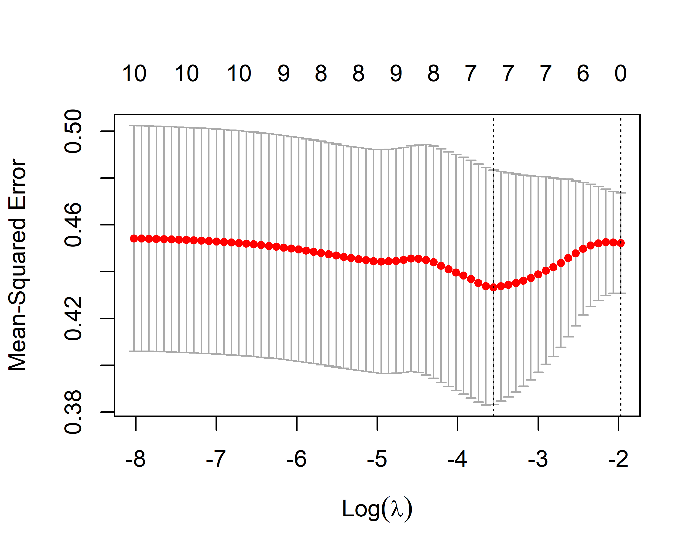


**Supplementary Figure S5.** **Feature selection process for constructing the diagnostic model using key metabolites**

(A, B) Feature selection is performed using the elastic net algorithm, with the optimal parameter selection process illustrated.


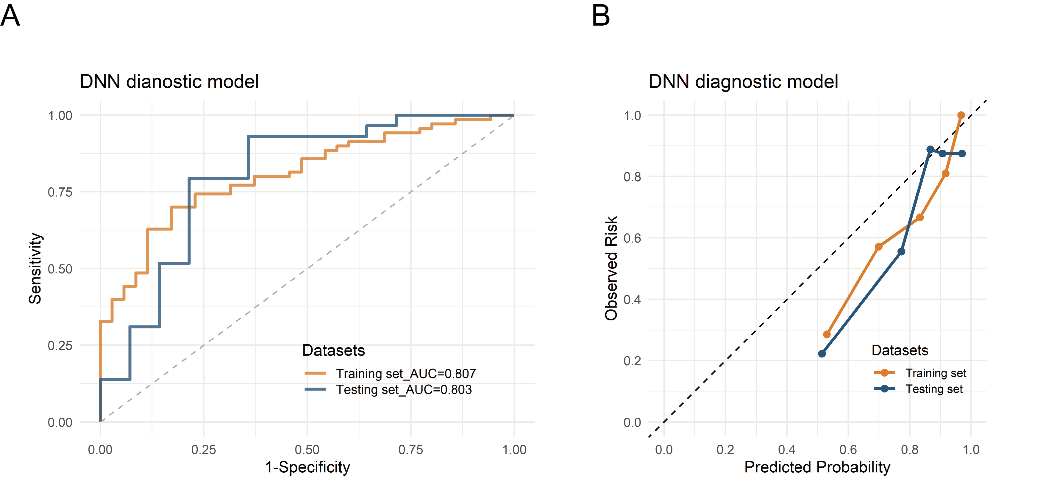


**Supplementary Figure S6.** **ROC and calibration curves of the DNN diagnostic model in the training and test sets.**

ROC, Receiver Operating Characteristic; DNN, deep neural network.
